# Combining Selective Internal Radiation Therapy (SIRT) and Systemic Therapy in Intrahepatic Cholangiocarcinoma

**DOI:** 10.3390/cancers18183017

**Published:** 2026-09-17

**Authors:** Francesco De Cobelli, Stephanie Steidler, Andrea Casadei-Gardini, Carla Canevari, Alessandro Loria, Francesca Ratti

**Affiliations:** 1 Department of Radiology, IRCCS Ospedale San Raffaele, 20132 Milan, Italy; 2 Vita-Salute San Raffaele University, 20132 Milan, Italy; 3 Department of Oncology, IRCCS Ospedale San Raffaele, 20132 Milan, Italy; 4 Department of Nuclear Medicine, IRCCS Ospedale San Raffaele, 20132 Milan, Italy; 5 Department of Medical Physics, IRCCS Ospedale San Raffaele, 20132 Milan, Italy; 6 Division of Hepatobiliary Surgery, IRCCS Ospedale San Raffaele, 20132 Milan, Italy

**Keywords:** intrahepatic cholangiocarcinoma, yttrium-90, selective internal radiation therapy, systemic therapy, downstaging

## Abstract

Background: Intrahepatic cholangiocarcinoma (iCCA) is the second most common primary liver cancer, with increasing presentation. Less than one-third of patients are resectable at diagnosis, and the first-line systemic standard, gemcitabine–cisplatin plus durvalumab, yields a modest median overall survival (OS) of approximately 13 months. In liver-dominant iCCA, selective internal radiation therapy (SIRT) with yttrium-90 (^90^Y) microspheres can deliver a high, parenchyma-sparing tumoricidal dose in loco and, paired with systemic therapy, is a valid treatment option. Methods: This commentary evaluates the retrospective and prospective data, comparative cohorts, registries, and meta-analyses of SIRT with systemic therapy in unresectable iCCA. Results: Across the data sets identified, the efficacy signal is in favor of combined approaches when SIRT is delivered in the first line selectively and concurrently with chemotherapy, reaching a median OS of about 22 months with 98% disease control. Propensity-weighted comparisons describe the added value of SIRT in improving OS and roughly doubling the progression-free survival and secondary resection rate. Downstaging to surgery (in approximately 5–19%) and, in highly selected patients with liver predominant disease, transplants can yield favorable longer-term survival with a good safety profile. Conclusions: To date, no completed randomized trial exists on combined locoregional SIRT and systemic therapy in patients with iCCA. SIRT should be considered a multimodal personalized therapeutic treatment option in patients with upfront unresecatable liver predominant disease.

## Simple Summary

Intrahepatic cholangiocarcinoma (iCCA) is a rare and aggressive disease which tends to be diagnosed once the malignancy is in an advanced stage. Surgery remains the curative treatment but only in a limited number of patients, and first-line treatment in unresectable disease is currently chemo- and immunotherapy. The aim of this commentary is to describe the combined effects of selective internal radiation therapy, also known as transarterial radioembolization with systemic treatment. The data available suggests that a combination of the two treatments in a defined sequential manner may ameliorate the outcomes of patients.

## 1. Introduction

Intrahepatic cholangiocarcinoma (iCCA) is a rare, aggressive malignancy arising from the bile duct epithelium proximal to the second-order ducts, with rising incidence in Western countries [1,2,3]. The disease tends to be diagnosed later or incidentally when lesions are larger in size, the disease is multifocal, or vascular invasion has occurred. Resectability applies to a limited number of patients, and 5-year recurrence-free survival ranges from 9% to 34% [1,4]. In patients that present with unresectable, liver-predominant disease, the therapeutic goal is to prolong survival with good quality of life, with a potential prospect of conversion to surgery.

The current first-line treatment in biliary tract cancers, cisplatin-gemcitabine (CisGem) plus durvalumab, was introduced after the results of the phase-three TOPAZ-1 trial [5], in which immuno-chemotherapy improved the median overall survival (OS) from 11.5 to 12.8 months (HR 0.80, *p* = 0.021), with a 24-month OS of 24.9% versus 10.4%. The benefit of CisGem durvalumab was confirmed (median OS 12.9 versus 11.3 months, HR = 0.76) [6], with a 3-year OS of 14.6% versus 6.9% [7] and comparable quality of life [8], as also reported by real world data [9,10]. The other approved first-line systemic treatment, pembrolizumab plus CisGem (KEYNOTE-966), provided a similar modest increase in OS [11]. This small increase in survival following systemic therapy defines the unmet need that liver-directed therapy seeks to address. Locoregional treatments such as TACE [12], ablation [13,14,15,16], and selective internal radiation therapy (SIRT) [12,17] have been described in iCCA. In SIRT, resin or glass microspheres traced with Yttrium-90 (^90^Y) exploit the arterial supply of lesions to deposit in situ focal radiation while sparing surrounding parenchyma.

This commentary describes the combined-treatment strategies, systemic treatment with SIRT, in iCCA.

## 2. Evaluation of Imaging and Response Assessment

Objective response to treatment is measured on imaging mainly using the Response Evaluation Criteria in Solid Tumors (RECIST) v1.1 [18] and modified-RECIST (mRECIST) [19,20], which are standardized criteria for solid tumors and viability-based criteria, respectively, also applied for iCCA [21]. Modified RECIST [21] and EASL response assessment [22] rely on enhancement rather than size and best capture SIRT’s effect. Anatomic size-based criteria tend to underestimate the response after SIRT, as the devascularized tumor involutes slowly and may transiently appear enlarged or to have enhanced rims (pseudo-progression) in the weeks following treatment and is not truthful in its response on the treated target lesion. According to the literature, the objective response rate (ORR) on contrast-enhanced CT (ceCT) is to the order of 19.6% by RECIST 1.1 versus 67% by mRECIST [23].

Key imaging biomarkers include tumor vascularity and the lesion size. Hypovascular lesions are more often associated with lymphatic, perineural, and biliary infiltration and with poor survival, whereas hypervascular tumors show less aggressive behavior with better outcomes [24]. Therefore, surrogate endpoints for the response to treatment, like the objective response rate (ORR), according to these criteria, should be considered. Functional imaging (diffusion-weighted MRI/ADC change or ^18^F-FDG-PET metabolic response) add more prognostic information during work-up. Radiomic signatures derived from pretreatment ceCT have been shown to stratify patients by response and survival versus conventional clinical and imaging variables, identifying candidates most likely to benefit from ^90^Y-SIRT [25].

## 3. Current Landscape

Clinical practice guidelines [1,26,27,28,29] indicate transarterial procedures as feasible and safe treatment alone or in combination with systemic treatment in patients with liver-predominant or limited extrahepatic disease, with low levels of evidence. Published data with chemo-immunotherapy and SIRT is incredibly limited [30], and data is predominantly derived from chemotherapy with SIRT.

The efficacy of SIRT combined with CisGem was reported in treatment-naïve patients with unresectable iCCA [31], with an ORR at 3 months of 39%, a disease control rate of 98%, median progression-free survival (PFS) of 14 months (8–17), and median OS of 22 months (14–52); nine patients were downstaged to resection.

Combined with a CisGem–capecitabine, ^90^Y-TARE [32] in locally advanced disease showed a complete or partial response in 38% and 46% of participants, with a median OS and PFS of 29 and 13 months, respectively, and roughly half of the cohort had surgical conversion.

A first-line approach with SIRT in an intention for treatment analysis [33] found a PFS of 6.6 months (95% CI: 2.5–9.8) and an OS of 13.6 months (95% CI: 5.4–21.6). Chemotherapy (16/24) after SIRT increased the PFS and OS to 9 months (95% CI: 3.2–13.1) and 21.6 months (95% CI: 7.3–25.2), but no participants were downstaged.

In the combined analysis of the prospective CIRT/CIRT-FR studies [34], the median PFS and OS (95% CI) varied significantly depending on the order of treatment. First-line SIRT with concurrent chemotherapy was significantly better than first-line SIRT alone over increasing lines of treatment (*p* < 0.001 among all), respectively PFS and OS 11.3 (6.1–14.0) and 32.5 (11.8–37.0) months versus 7.4 (3.9–11.0) and 16.2 (9.0–27.7) months followed by second line SIRT with an OS of 5.1 (3.1–6.4) and PFS of 12 (8.2–20.8) months and gretaer than second line OS at 3.5 (2.5–4.3) and PFS of 9.3 (4.5–14.7) months.

A long-term real-world series [35] showed a median OS of 17 months (95% CI: 13.15–20.57), PFS of 7.5 months (95% CI: 5.00–9.50), a target lesion ORR rate of 40%, and a disease control rate (DCR) of 97%. The independent predictors were SIRT as the first-line treatment (HR = 2.06, *p* = 0.008) and low tumor volume (<150 mL; HR = 1.96, *p* = 0.011).

The first-line treatment effect increased the median OS in a large retrospective cohort [36]. Longer survival was recorded in mass-forming tumors versus infiltrative disease at 12.0 months (95% CI: 9.5–15.9) and 7.6 months (95% CI: 5.8–12.7) (*p* = 0.038), respectively. Hypervascular tumors responded better than hypovascular or mixed lesions, with a median OS of 12.7 (95% CI: 9.8–17.0); 8.0 (95% CI: 5.4–12.0), and 9.1 months (95% CI: 8.1–16.9), respectively (*p* = 0.037), and distant metastases predicted a shorter OS (*p* = 0.004).

Chemo-naïve patients receving SIRT in the ReSIN study [37] had a median OS of 10.6 months (95% CI: 3.5–16.9) versus 19.1 months (95% CI: 12.3–26.0) in those who had recieved prior chemotherapy (*p* = 0.07), potentially a selection effect rather than a treatment effect. The OS for participants with radiological ORR versus stable or progressive disease at 3 months was 12.2 (95% CI: from 7.9 to NR) versus 12.5 (95% CI: 4.3–26.1) months, and patients with a 6-month ORR had an OS of 16.5 months, with a significant hazard ratio (*p* = 0.008).

A propensity-matched analysis of a database cohort [38] reported substantially longer survival in SIRT-treated patients (matched median OS: 17.5 versus 7.2 months), with better outcomes when administered prior to surgery or associated with systemic chemotherapy. In a propensity-weighted (target-trial emulation) analysis [39], SIRT with systemic themotherapy showed a median OS of 21.7 (14–52) versus 15.9 months (HR 0.59, *p* = 0.049) in those with chemotherapy alone and a median PFS of 14.3 (8–17 months) versus 8.4 months (HR 0.52, *p* < 0.001), respectively, in favor of the combined treatment strategy with an unadjusted odds ratio for conversion to surgery of 3.87. Pooled weighted analysis previously reported data [17] showed a 23.4% response rate with a 7.8-month PFS and 14.1-month OS in the sub-cohort treated with SIRT in which 60% (469/782) of patients had received chemotherapy.

TARE plus chemotherapy, compared with chemotherapy alone [40], showed an unadjusted median OS of 22.5 versus 15.1 months (95% CI: 0.57–1.01; HR 0.76) and a PFS of 10.8 versus 5.5 months (95% CI: 0.41–0.71, HR 0.54, *p* < 0.0001), respectively. The ORR was 58% versus 28%, and the secondary resection rate was 18.7% versus 8.8%. Both roughly doubled with comparable toxicity (ORR-OR: 3.17, 95% CI: 2.18–4.49, *p* < 0.0001; OR: 2.94, 95% CI: 1.71–5.03, *p* < 0.0001) after inverse-probability weighting. The maximum benefit in terms of response was observed when SIRT was delivered approximately within two months of starting systemic therapy.

A metanalysis reported a disease control-rate of 82%, PFS of 7.8 months, and median OS of 12.7 months, with successful downstaging in 11% of patients (921 patients in 21 studies) [41].

A mean OS of 15.8 months and 1-, 2-, and 3-year survival rates of 52.6%, 27%, and 16.8% were extrapolated from 1365 patients in 27 studies, respectively, with treatment-naïve or first-line patients having the better OS (19.7 versus 12.2 months) [23]. The authors also reported that surgical downstaging (pooled rate of 4.9%; range of 3–54% across studies) was associated with a mean survival of about 35 months and with a pooled ORR 19.6% with RECIST 1.1 versus 67% with mRECIST. Naïve mass-forming tumors with a low burden benefitted the most from this approach, with an estimated median OS of 19.3 months for peripheral versus 8.2 months for infiltrative iCCA [42]. The two large meta-analyses provide meaningful pooled estimates lacking randomized trials. 

The main studies are summarized in Table 1.

## 4. Integrated Approach: Prognostic Factors, Dosimetry, and Molecular Profile

Recurring prognostic factors across studies include the performance status, tumor burden and distribution, vascular involvement, histological differentiation, and the presence of extrahepatic disease. Baseline ascites and a poorer performance status predicted a lower OS and PFS [37]. The absence of extrahepatic disease and the absence of ascites are predictors of progression-free survival [34], and ECOG 0–1 correlates with a better median OS [32,43]. Treatment-naïve first-line SIRT yields better survival and downstaging rates [23]. Inflammatory markers, such as the neutrophil-to-lymphocyte and platelet-to-lymphocyte ratios, have also been investigated as candidate biomarkers of outcomes [44].

Personalized dosimetry planning significantly outperformed previously used body surface area methods [26]. Voxel-level dosimetry and dose–volume metrics (e.g., D70) better capture dose heterogeneity and correlate more accurately with biological responses than the average tumor dose [45], which becomes particularly critical in iCCA, with the peripheral-dominant microsphere deposition that traditional average-dose models fail to characterize. Partition-model dosimetry independently predicted an improved OS (HR = 0.59, 95% CI: 0.37–0.94, *p* = 0.026) [34], and the MIRD model led to high rates of tumor necrosis and R0 resections [46]. Intrahepatic CCA-specific dose thresholds are still largely extrapolated from HCC (tumor absorbed dose ≥205 Gy, normal liver ≤75 Gy, and ≥400 Gy for radiation segmentectomy), yet the ORR was near 94%, and a dose response for complete necrosis >180 Gy was proposed [47,48] for glass microspheres. Voxel-based thresholds for resin microspheres may target lower tumor-absorbed doses due to differing embolic loads and particle characteristics [49].

Treatment with 90-Y glass or resin microspheres is extremely selective; dose deposition directly targets the tumor and perfused tissue without toxicity to the non-perfused liver volume. Real-world multivariable data indicates that selectivity of delivery may outweigh the need for a single-dose threshold, refocusing the technique on targeted, parenchyma-sparing infusion [35].

PET-based dosimetry in resin Y-90 spheres showed a significant correlation to the mean tumor-absorbed dose (TAD) and ORR (*p* = 0.020, AUC = 0.693) [50] compared with post-treatment ^90^SPECT. A higher TAD was correlated to the ORR (320 versus 166 Gy median dose) in patients treated with resin microspheres on a Y-90 PET–based dosimetry (*p* = 0.008), with a cut-off mean value of 298 Gy (specificity = 91%; sensitivity = 55%).

Higher doses and activity were correlated to “extensive necrosis” (≥90%), with 200 Gy compared with 120 Gy for non-extensive necrosis and 2.0 GBq versus 1.1 GBq, in a small cohort receiving ^90^Y resin SIRT followed by surgery [51]. The authors reported that a dose ≥180 Gy had a significant probability to achieve extensive necrosis (odds ratio = 13.3, *p* = 0.046) versus lower doses. In this cohort, all lesions with extensive necrosis (*n* = 3) had PR via RECIST, whilst seven lesions had CR via mRECIST. Naïve subjects treated with SIRT had more extensive necrosis that those who had received chemotherapy (10/11 versus 3/7; Fischer’s *p* = 0.047), with a significant difference between the activity, dose, and mRECIST CR-predicted extensive necrosis (100% PPV and 44% NPV).

In iCCA, “actionable” alterations described in molecular profiling may increase outcomes, as described by Job et al. [52], who identified four distinct tumor microenvironment subtypes in iCCA which may direct even more personalized immune-based therapies. Molecular profiling of a large patient cohort showed that IDH1 and ARID1A alterations were the most frequently detected, occurring in approximately 20% of cases, while TP53/CDKN2A, FGFR2, and KRAS alterations were reported in 17%, 15%, and 10% of patients, respectively [53]. Among individuals with unresectable disease, TP53, KRAS, and CDKN2A alterations were associated with a particularly unfavorable prognosis and were therefore considered “high-risk signatures.” Oncogenic variants confirmed in iCCA in a recent review [54], namely HER2, IDH1, FGFR2, BRAF, NTRK, and RET, may be used to further target a choice in systemic treatment. SIRT is known to locally induce an inflammatory tumor microenvironment with recruitment and activation of immune cells, both local and systemic, as described in hepatocellular carcinoma [55,56], in which distinct immune profiles may identify responders. Unresectable iCCA treated with SIRT also modifies the immune profiles of extravesicular vesicles [57], suggesting that combined immunotherapy with SIRT may ameliorate outcomes and increase resectability.

## 5. Safety of SIRT and Combined Treatments

The most common treatment related complication after SIRT is post-embolization syndrome characterized by grade 1–2 fatigue, nausea, low-grade fever, and abdominal pain due to locoregional inflammation within 72 h of the procedure [58,59], ranging from 20% to 55% in patients with HCC [59] and cellular reactions. More prolonged manifestations, such as radiation-induced (beta) hemolysis and erythrocyte suppression in the myeloid tissues, are rare and can lead to anemia and leukopenia [58]. Severe complications following neoadjuvant TARE with resin microspheres were reported to be approximately 6% according to Sawar et al. [46], and Cocozza et al. [23] reported 45.7% of patients having adverse events, of which only 5.9% were severe.

Delayed effects may include radiation-induced grade 1–2 cytopenia [58] in approximately 10% of patients [45], as well as anemia and leukopenia [58]. In a combined series [34], the overall toxicity was mild (abdominal pain 19.5%, nausea 17.8%, and fatigue 30%); radioembolization-induced liver disease (REILD) was present in 2.3% of patients. Mild events, such as abdominal pain (18.1%), fever (18.1%), fatigue, and vomiting, were also described in another cohort [35], in which no event above grade 3 occurred after 30 days from SIRT. Severe complications are uncommon, increasing with extensive disease and high administered doses.

Concomitant systemic therapy may cause hematological toxicity, as observed in the MISPHEC participants [31], in which mostly grade 3–4 chemotherapy events were recorded in 29/41 patients while maintaining a good quality of life. The event frequency is similar to those described in the immuno-chemotherapy arm of the TOPAZ-1 study, where 75.7% of patients had any-cause grade 3–4 events, of which 62.7% were treatment-related [5]. Adjustments to reduce SIRT-related toxicity include the use of multicompartment dosimetry which spares normal livers, ^99m^Tc-MAA mapping to exclude non-target and gastrointestinal flow, abiding or being more conservative to the maximum lung shunt fraction, and antibiotic prophylaxis in the presence of biliary stent or bilio-enteric anastomosis, a principal risk factor for post-procedural cholangitis or abscess [60,61].

TARE plus first-line immuno- or chemotherapy improves outcomes [34,36,38,39], with limited and mostly manageable events.

## 6. Downstaging to Surgery and Bridging to Transplant

Converting initially unresectable disease to resection or transplant is the desired outcome of any treatment scheme in iCCA [29]. Transarterial radioembolization also induces atrophy of the treated lobe with compensatory contralateral hypertrophy (“radiation lobectomy”), which can increase the future liver remnant and support a bridge-to-surgery strategy [62]. SIRT plus systemic chemotherapy for downstaging in upfront unresctable disease has been described and proposed [63,64]. In a multivariable analysis [65], SIRT as downstaging intervention was independently associated with a better OS (HR = 0.34, 95% CI: 0.14–0.84, *p* = 0.019) and more effective than chemotherapy [65] . Adamus et al reported an increase in resection rates for combined SIRT and chemotherapy (18.7% versus chemiotherapy alone 8.8%, *p* = 0.0279), confirmed after adjustment for confounding factors (IPTW) (OR = 2.94, 95% CI: 1.71–5.03, *p* < 0.0001) [40].

Conversion rates reported in Cocozza et al.’s metanalysis [23] were in the range of 3–54% (summary proportion). and those who received surgery had an increased 1-year survival (meta-regression analysis, OR = 8.25, 95% CI: 1.58–42.91, *p* = 0.01). Yu et al. [66] described that SIRT alone or combined with systemic treatment was associated with a high percentage of tissue necrosis which in turn was associated with an ameliorated median survival time after surgery or transplant [66].

Resection following SIRT plus chemotherapy, increased the 12- and 24-month PFS rates to 66.7% (95% CI: 35.9–97.5%; 95% CI: 35.9–97.5%) which was maintained at both of these timepoints and the 12-month OS rate to 88.9% (95% CI: 68.4–100%) [31]. 

Downstaging led to transplants in 4/13 (31%) participants, as described in a single-arm intention-to-treat study [67] in which SIRT was administered after four cycles of chemotherapy, with a DFS between 8 and 73 months. Six patients under 70 years of age (within a larger cohort) [68] were transplanted following neoadjuvant treatment and SIRT between cycles 2 and 3 (19 were resected), with a 5-year OS of 100% and 5-year PFS of 44.4% after transplantation, versus an OS of 45.3% after secondary resection and 25.9% with neither (*p* = 0.03).

These outcomes were derived from small, highly selective groups in which early progression and comorbidities excluded most candidates. Compensatory hypertrophy of the contralateral parenchyma following SIRT mimics portal-vein embolization and increases the future liver remnant [64]. However, measurable volume gain alone does not guarantee resection as tumor biology and clinical status may determine outcomes, and only a minority ultimately proceed to surgery.

## 7. Clinical Trials and Future Trends

Some of the clinical trials investigating combination treatment published in clinicaltrials.gov have been terminated or suspended. The randomized SIRCCA trial [69] was designed to evaluate the effect of combined SIRT and chemotherapy versus chemotherapy alone was terminated due to slow accrual. The other randomized trial, SIROCHO, with neoadjuvant glass ^90^Y plus capecitabine followed by surgery versus upfront surgery, was stopped and reactivated recently in August 2025 [70], and it aims to provide the effects of neoadjuvant treatment in terms of participants with adequate surgical margins. 

The ongoing trials that combine TARE with chemo-immunotherapy are listed in Table 2. The details in the table are those available and extrapolated from the NCT website. The rationale for combining SIRT with immunotherapy, in addition to the direct cytotoxicity, lies in the radiation-induced necrosis which releases tumor antigens and generates an acute inflammatory tumor microenvironment, with recruitment and activation of immune cells as described in hepatocellular carcinoma [55]. 

The IMMUWHY (NCT04238637) study administers SIRT in advanced stage unresectable patients first and then randomizes participants into one the two immunotherpay arms, durvalumab alone or with temelimumab with ORR as primary endpoint. The trial conducted at Beth-Israel Deaconess Medical Center (NCT05655949) is a single arm study in patients with advanced unresectable or metastatic iCCA that administers SIRT between cycle 2 and 3 of chemo-immunotherpay with CisGem and durvalumab for 8 cycles with PFS as primary endpoint. The PM-CARE trial (NCT06375915) investigates upfront ^90^Y-SIRT in unresectable, liver-predominant iCCA followed by six cycles of cisplatin–gemcitabine plus durvalumab. The primary endpoint is objective response according to mRECIST at 6 months. The study design is based on the rationale of a priming effect in situ immune response, which seems most effective in treatment-naïve patients when upfront surgery is not an option [71].

Randomization against systemic therapy or against other locoregional techniques alone of in combination is needed to establish the role of SIRT in iCCA and as a standard of care, yet such trials may be difficult to complete. [72].

Adjunct combined treatment strategies may come from complementary and alternative medicines mostly used in Asian countries. In patients with hepatocellular carcinoma, these were administered with transarterial chemoembolization [73], radiofrequency [74], or in combination with systemic standard of care [75], ameliorating the outcomes, yet limited data exists for cholangiocarcinoma [76]. In vitro data [77] has shown the immunomodulatory effects of plant extracts and their active component and downregulation in vitro and in animal models of liver fibrosis [78]. These treatments may augment effects induced by SIRT locally by modulating the tumor microenvironment and inhibiting angiogenesis but may not be adaptable to all populations. 

## 8. Discussion

The current first-line standard of care in advanced, unresecatbleor metastatic iCCA is chemotherapy with cisplatin and gemcitabine plus durvaulamb or pembrolizumab i. and available evidence supports the added value of combined approaches with SIRT in these patients . Prospective data reports a median OS of more than 20 months, and comparative and propensity-weighted data show an improved OS and roughly doubled PFS, with ameliorated secondary resection rates. 

In the absence of randomized clinical trial data, real-world multivariable analyses point to timing and selectivity to achieve better outcomes. SIRT adds value to systemic therapy and is more effective when delivered first and selectively (personalized dosimetry) to low-volume targets, maintaining adequate liver function and quality of life, and it is also being considered as an early option in multimodal therapy, mimicking treatment strategies in HCC.

Responses should be evaluated a considering also enhancement accoridng to mRECIST or EASL and functional imaging rather than the size criteria alone to avoid mistaking slow involution or transient swelling as treatment failure, considering the complexity of the overall disease, and include specific molecular profiling for targeted immunotherapy [79].

The conversion endpoints after a combined therapeutic regimen, resection and for a select few transplants, are of high-value and achievable (up to 52%) [42,46,65], even though this is only for a minority of patients [67]. Real-world data for patients treated with CisGem plus durvalumab [80] showed that 11.8% of patients with locally advanced disease received surgery, suggesting an important role for SIRT in increasing resectability.

Administered early and selectively in a multidisciplinary pathway, SIRT can be a valuable treatment option in addition to systemic therapy in personalized strategies. The principal limitation of data available is the quality of produced evidence; data is dominated by small, heterogeneous cohorts with varied disease stages, tumor types, dosimetry, and treatment sequences. The two randomized trials that would have provided definitive answers were or stopped early or were put on hold, and evaluation is susceptible to selection and reporting bias. 

## 9. Conclusions

Overall, SIRT with Yttrium-90 is a safe and effective option in unresectable, liver-predominant iCCA, in which personalized dosimetry and selective delivery are important for outcomes. The combined strategy has its greatest benefit in first-line, selective, low-volume lesions and when combined with immune- or chemotherapy. Adequately powered randomized trials should be the priority for producing quality evidence, but low numbers and heterogenous populations may support high-volume real-world studies for investigating combined treatment schemes.

## Figures and Tables

**Table 1:** Selected works for SIRT ± systemic therapy in unresectable iCCA.

Study and Design	*n*	Treatment and Timing	Response and Disease Control	Median PFS (mo)	Median OS (mo)	Conversion to Surgery
Edeline et al. (MISPHEC) [31] Prospective, multicenter, single-arm phase II	41	Y-90 glass SIRT + Gem-Cis; first-line, concomitant	RECIST 1.1: ORR = 39%; DCR = 98%	14 (8–17)	22 (14–52)	22%; 8 R0 resections
Ahmed et al. [32] Retrospective, single-center study	13	Y-90 SIRT + Gem-Cis-Cape; first-line, concomitant	mRECIST: CR 38.5%; PR = 46.2%	13	29	53.8%
Chan et al. [33] Prospective, multicenter, single-arm phase II	24	Resin Y-90 SIRT + Gem-Cis; first-line, sequential	RECIST 1.1: ORR = 25%; DCR = 75%	6.6 * 9.0 ^‡^	13.6 * 21.6 ^‡^	not achieved
Edeline et al. [39] Propensity-weighted comparative analysis	114 ^§^	Y-90 SIRT + chemotherapy Vs. chemotherapy alone; first-line	NR	14.3 vs. 8.4	21.7 vs. 15.9	22% vs. 4% (*p* = 0.062) Adjusted 8% vs. 3% (*p* = 0.113)
Adamus et al. (Real-SIRTCCA) [40] Real-world comparative study	277	TARE + chemotherapy Vs. chemotherapy alone; concurrent treatment	RECIST 1.1: ORR = 58% vs. 28%	10.8 vs. 5.5	22.5 vs. 15.1	18.7% vs. 8.8%
Reimer et al. (CIRT/CIRT-FR) [34] Prospective, multicenter observational study	20	Y-90 TARE + chemotherapy; first-line, concurrent	Outcomes varied according to treatment line; best outcomes reported for first-line concurrent treatment	11.3	32.5	NR
Bracchischi et al. [35] Retrospective, real-world study	72	Resin Y-90 TARE with or without chemotherapy; first-line or subsequent-line treatment	RECIST 1.1: ORR = 40%; DCR = 97%	7.5 (5–10)	17 (13–21)	NR
Schartz et al. [41] Systematic review and meta-analysis	921 Pooled 21 studies	Y-90 TARE across 21 studies; heterogeneous treatment strategies and timing	DCR = 82%	7.8	12.7	11%
Cocozza et al. [23] Systematic review and meta-analysis	1365 Pooled 27 studies	Y-90 TARE ± systemic therapy; heterogeneous treatment strategies and timing across included studies.	RECIST 1.1 ORR = 19.6%; mRECIST ORR = 67%	15.8	at 1, 2, 3 yrs 53, 27, 17%, respectively	4.9%

* ITT population; ^‡^ Post-TARE chemotherapy subgroup (*n* = 16). ^§^ In all, 41 patients received SIRT + chemotherapy and 73 chemotherapy alone; outcomes from propensity-weighted analysis. **Abbreviations:** DCR = disease control rate; Gem-Cis = gemcitabine plus cisplatin; mo = months; NR = not reported; ORR = objective response rate; OS = overall survival; PFS = progression-free survival; RECIST = Response Evaluation Criteria in Solid Tumors; SIRT = selective internal radiation therapy; TARE = transarterial radioembolization; Y-90 = yttrium-90.

**Table 2:** Ongoing clinical trials that combine SIRT or TARE with chemo-immunotherapy (from NCT website).

NCT Number	NCT Title	Trial Design	Treatment Strategy	Primary Endpoint	Status (Last Update)
NCT04238637	Immunotherapy Combined With Y-90 SIRT Therapy in Advanced Stage Intrahepatic Biliary Tract Cancer IMMUWHY	Multicenter Phase II, randomized, prospective, open-label Trial 2 arms: SIRT + Durvalumab and Tremelimumab and SIRT + Durvalumab	SOC SIRT plus systemic immunotherapy	ORR by RECIST 1.1 up to 20 months	Active, not recruiting (5 December 2025)
NCT05655949	Y-90 With Durvalumab/Gem/Cis in Intrahepatic Cholangio	Single-arm, phase-2 study ^90^Y-SIRT with durvalumab and gemcitabine–cisplatin in locally advanced unresectable or metastatic intrahepatic cholangiocarcinoma.	Chemo- immunotherapy for 8 cycles Q21days week 2 or 3—one-time 90-Y SIRT ≥9 cycles Durvalumab maintenance	Median progression-free survival (PFS) RECIST 1.1 Incidence of Grade 3 or higher treatment-related toxicity	Recruiting (22 April 2026)
NCT06375915	Precision Medicine in Patients with Unresectable Cholangiocarcinoma: Radioembolization and combined biological therapy	Multicenter, Phase II, single-arm Upfront unrescetable liver predominant iCCA 90-Y TARE plus cisplatin/gemcitabine and durvalumab	90-Y SIRT followed by 6 cycles Q21 days of durvalumab + cis/gem	ORR at 6 months Safety number of participants with treatment-related; adverse events	Recruiting (2 September 2026)
NCT06910722	Liver Transplantation for Locally Advanced Intrahepatic Cholangiocarcinoma After SIRT and Chemotherapy (RIS-TH)	Phase 2 study Single arm	SIRT + chemotherapy improves 3-year OS after transplant in locally advanced intrahepatic cholangiocarcinoma (unresectable but not metastatic)	OS at 3 years after transplant	Recruiting (6 April 2026)
NCT06862934	Liver Transplantation for Unresectable Intrahepatic Cholangiocarcinoma After Sustained Response to Neoadjuvant Treatments (iCOLA)	Single-arm, observational cohort study	Efficacy and safety of neoadjuvant downstaging strategies that include 90-Yttrium-TARE with chemotherapy +/− immunotherapy	OS at 3 years after liver transplant	Recruiting (6 March 2025)
NCT07043387	Streamlining Radioembolization for CCC and Metastatic Liver Cancer (ISTAR-03)	Observational, multicenter, prospective registry, case-only	SIRT; observational nature may include following treatments	ORR by RECIST up to 12 months	Recruiting (18 August 2026)
NCT05714124	Liver Embolization Approaches for Tumor Management (LEATUM)	Observational, single-center, prospective, and retrospective study in primary and secondary lover tumors	Embolization techniques including TARE with or without systemic treatment	PFS at 3 and 6 months (short term), at 1 year (mid), up to 5 years (long term)	Recruiting (12 April 2024)

## Data Availability

Data cited is publicly available.

## Abbreviations

The following abbreviations are used in this manuscript:

iCCA	Intrahepatic cholangiocarcinoma
^90^Y	Yttrium-90
HR	Hazard ratio
OS	Overall survival
SIRT	Selective internal radiation therapy
ORR	Objective response rate
PFS	Progression free survival
RECIST	Response Evaluation Criteria in Solid Tumors
EASL	European Association for the Study of the Liver
